# Out-of-Plane Mechanical Behavior of 3D-Printed Polymeric Circular-Vertex-Based Hierarchical Hexagonal Honeycombs

**DOI:** 10.3390/polym17070862

**Published:** 2025-03-24

**Authors:** Yong Tao, Xiyu Chen, Siping Lu

**Affiliations:** School of Civil Engineering, Central South University, Changsha 410075, China; tao-yong@csu.edu.cn (Y.T.); 234811008@csu.edu.cn (X.C.)

**Keywords:** hierarchical hexagonal honeycomb, experimental investigation, out-of-plane, mechanical properties, energy absorption

## Abstract

Many studies show that hierarchical honeycombs have a superior performance compared to regular honeycombs. However, relevant experimental studies are limited due to the fabrication challenges of hierarchical honeycombs featuring complex geometries. In this study, circular-vertex-based hierarchical hexagonal honeycombs (CHHHs) with different hierarchical parameters were fabricated using a polymeric 3D-printing technique, and their quasi-static out-of-plane mechanical behavior was investigated. The CHHHs were constructed by replacing each solid vertex of a regular hexagonal honeycomb (RHH) with a circular vertex. Quasi-static compression tests were conducted on CHHHs, and the effect of the hierarchical parameter on the deformation modes, mechanical properties, and energy absorption characteristics was investigated. The results revealed that both the CHHH and RHH specimens experienced cell wall fractures during compression, while the CHHH exhibited enhanced damage resistance, compressive strength, and specific energy absorption (SEA) compared to RHH. This study contributes to understanding the effect of circular-vertex-based hierarchy on the out-of-plane mechanical behavior of regular honeycombs.

## 1. Introduction

Honeycomb materials are widely utilized in load-bearing and energy absorption applications due to their exceptional specific stiffness and strength and remarkable energy absorption capability [1]. Researchers have conducted extensive studies on the in-plane [2,3,4] and out-of-plane [5,6,7] mechanical behaviors of regular honeycombs with various configurations. Their findings indicated that the out-of-plane mechanical properties (e.g., stiffness, strength, etc.) of regular honeycombs were superior to their in-plane mechanical properties. Moreover, it was also found that the mechanical properties of honeycombs can be significantly influenced by their cross-sectional configurations [8,9]. Given that honeycombs are frequently employed as core materials in sandwich panels that primarily bear out-of-plane loads [10], there is a long-term effort to develop novel configurations that enhance the out-of-plane mechanical properties of regular honeycombs.

Through a long period of evolution, biological materials have developed many excellent mechanical properties, such as lightweight, high strength, and superior impact resistance [11], and their hierarchical structures have been demonstrated to be a critical factor contributing to these advantages [12,13]. Inspired by the hierarchical structures in natural biological materials, hierarchical design is also employed in honeycomb materials to enhance their mechanical properties. By replacing the solid cell walls of regular honeycombs with cellular materials or structures, various innovative hierarchical honeycombs with superior mechanical properties have been designed [14,15,16,17]. For example, Chen and Pugno [18] constructed a new class of hierarchical honeycombs, and theoretical analyses revealed that their elastic buckling properties can be tailored at different hierarchical levels. Two kinds of anisotropic multifunctional hierarchical honeycombs (AMHHs) were designed by Sun et al. [19], and the results indicated that the in-plane stiffness of AMHHs can be more than 100 times that of the corresponding anisotropic hexagonal honeycomb. Fang et al. [20] introduced structural hierarchy into a regular hexagonal honeycomb (RHH) by replacing each hexagonal cell side with hexagons, and they found that the second-order hierarchical honeycomb achieved a plateau stress 2.63 times higher than the RHH. By replacing the sidewall of a regular square honeycomb (RSH) with square honeycomb core sandwich panels, Fan et al. [21] constructed hierarchical lattice structures with an extraordinary energy absorption ability. Similarly, a bi-factorial hierarchical honeycomb was proposed based on RSH, and its efficient progressive folding deformation along with promising out-of-plane crushing resistance was revealed by Huang et al. [22].

By replacing the vertices of an RHH with smaller hexagons, Ajdari et al. [23] proposed a vertex-based hierarchical honeycomb, and it was found that the first- and second-order hierarchical honeycombs were 2.0 and 3.5 times stiffer than the equal-mass RHH. Afterward, a series of vertex-based hierarchical honeycombs were constructed by replacing the vertices of regular honeycombs with polygons such as triangles, squares, and circles [24,25]. For example, Hua et al. [25] proposed a hierarchical triangular honeycomb by repeatedly replacing the vertices of an RHH with equilateral triangles, and excellent energy absorption was achieved. By replacing each vertex of an RSH with a smaller square, Wang et al. [26] designed a vertex-based hierarchical honeycomb and found its folding response under compression was more stable than that of the RSH. Similarly, Chen et al. [27] and Wu et al. [28] designed hierarchical circular-joint honeycombs by replacing the vertices of an RHH and RSH with circles, respectively, and investigated their in-plane mechanical properties and out-of-plane crashworthiness. More recently, by replacing the vertices of the edge-based hierarchical square honeycomb [1,29] with smaller diamonds, Tao et al. [30,31,32] proposed a novel hybrid hierarchical square honeycomb (HHSH). According to the results, the HHSH exhibited a wide range of tailorable in-plane Young’s moduli [30,32], and its out-of-plane plateau stress could be enhanced by 223% compared to the corresponding RSH [31]. In addition, numerous excellent studies have been conducted on vertex-based hierarchical honeycombs, and various unique mechanical properties have been found, such as high stiffness [33,34,35], a negative Poisson’s ratio [36,37], high strength [38,39,40], and excellent energy absorption [41,42,43,44].

Currently, there are many innovative investigations into hierarchical honeycombs. However, most of the investigations are conducted using numerical and theoretical methods, and limited experimental studies have been reported, primarily due to the complex geometry of hierarchical honeycombs and the challenges associated with their fabrication using traditional methods [1,25]. Therefore, this study aimed to further investigate the unique mechanical behavior of hierarchical honeycombs under out-of-plane compression through experimental tests. The rest of this article is organized as follows. Section 2 describes the geometry of the studied hierarchical honeycomb and derives its relative density. Section 3 introduces the fabrication and testing details of the hierarchical honeycomb. Section 4 discusses and analyzes the deformation mode, mechanical properties, and energy absorption characteristics of the hierarchical honeycomb. In Section 5, the main conclusions of this study are summarized.

## 2. Geometric Description of CHHHs

**Figure 1:**
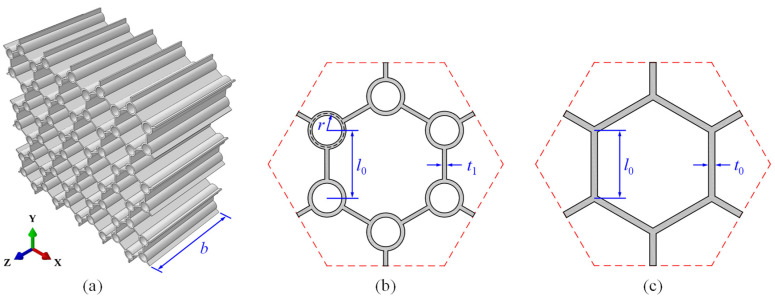
(**a**) Three-dimensional schematic diagram of CHHH, and unit cell cross-sections of (**b**) CHHH and (**c**) RHH.

Figure 1 illustrates the three-dimensional schematic diagram of the circular-vertex-based hierarchical hexagonal honeycomb (CHHH, Figure 1a), as well as the unit cell cross-sections of the CHHH (Figure 1b) and regular hexagonal honeycomb (RHH, Figure 1c). It can be seen from Figure 1b,c that the CHHH is constructed by replacing each solid vertex of an RHH with a circular vertex. For the CHHH, *t*_1_ and *r,* respectively, denote the cell wall thickness and radius of the circular vertex, and the distance between the centers of two adjacent circular vertices is *l*_0_, as shown in Figure 1b. For an RHH, the cell wall thickness and side length are, respectively, denoted as *t*_0_ and *l*_0_, as shown in Figure 1c. The overall dimensions of a CHHH are $L_{x}\times L_{y}\times b$, where *L_x_* and *L_y_* are the length and width along the *x* and *y* directions (Figure 1a), respectively, and *b* is out-of-plane thickness along the *z* direction. To characterize the vertex-based hierarchy of CHHH, the hierarchical parameter $R=r/l_{0}$ is defined. By considering the constraint of avoiding geometric overlapping, the hierarchical parameter *R* of the CHHH should satisfy the constraint of $0\leq R\leq \left(1-t_{1}/l_{0}\right)/2$, where the cell wall thickness *t*_1_ is taken into account.

**Figure 2:**
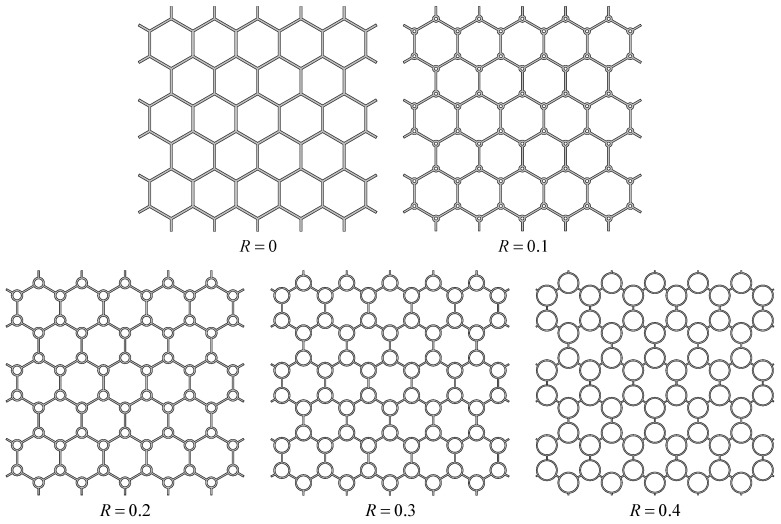
Cross-sections of CHHHs with different hierarchical parameters.

To illustrate the meaning of hierarchical parameter *R* clearly, the cross-sections of CHHHs with different *R* are presented in Figure 2. It can be seen that the size of the circular vertex in CHHH increases with the increase in *R*. Meanwhile, it is noted that the CHHH degrades to an RHH when R=0. According to the geometries of the CHHH and RHH, their relative densities can be expressed as follows:

(1) $${\bar{\rho }}_{\mathrm{CHHH}}=\frac{2}{\sqrt{3}}\frac{t_{1}}{l_{0}}\left[1+\left(\frac{4\pi }{3}-2\right)R-\frac{t_{1}}{l_{0}}\right]$$

(2) $${\bar{\rho }}_{\mathrm{RHH}}=\frac{2}{\sqrt{3}}\frac{t_{0}}{l_{0}}\left(1-\frac{1}{2\sqrt{3}}\frac{t_{0}}{l_{0}}\right)$$

Note that the cell wall thickness is considered to derive the above two formulas. When the ratios $t_{1}/l_{0}$ and $t_{0}/l_{0}$ of honeycomb materials are very small, their quadratic terms can be neglected [1,27], and then the above formulas for the relative densities of the CHHH and RHH can be degraded to those in the previous study [24].

## 3. Experimental Test

### 3.1. Specimen Fabrication

**Figure 3:**
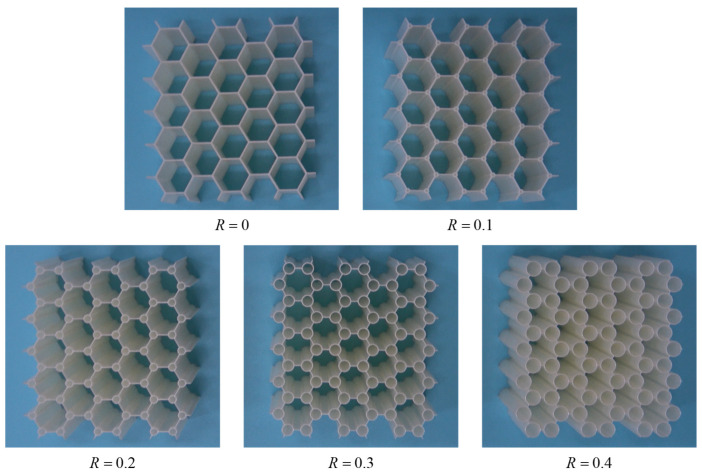
Typical specimens of CHHHs with different hierarchical parameters.

Figure 3 presents the specimens of CHHHs with different hierarchical parameters. These specimens were fabricated using a commercially available 3D printer Objet350 Connex3™ (Stratasys Ltd., den Prairie, MN, USA). The printer has a dimensional accuracy of ±100 µm for models under 100 mm and ±200 µm or ±0.06% of the length for larger models. Utilizing PolyJet technology with 600 dpi (*X*/*Y*-axis) and 1600 dpi (*Z*-axis) resolutions, it can achieve a layer thickness as fine as 16 µm, which enables precise fabrication of complex geometries such as hierarchical honeycombs. For comparison, all specimens have the same overall dimensions, which are 77.21 mm×72 mm×40 mm, and their masses are also kept the same. The cell wall thickness of the RHH specimen (R=0) is $t_{0}=0.75\;\mathrm{mm}$, and the side length is $l_{0}=8\;\mathrm{mm}$. Therefore, according to Equation (2), the relative density of the RHH specimens can be obtained as 0.1053. Using the relative density, *R*, and *l*_0_, the cell wall thickness *t*_1_ of the CHHH can be calculated from Equation (1). The geometric dimensions of the CHHH specimens with different *R* are summarized in Table 1. It can be found from the table that the cell wall thickness *t*_1_ of the CHHH decreases with the increase in *R* as a result of maintaining the same relative density $\bar{\rho }$. The base material of the fabricated CHHH specimens was a rigid polymer (VeroWhitePlus). The tensile specimens of the base material were printed and tested in our previous study [1]. According to the tensile test results, the basic mechanical parameters of VeroWhitePlus are as summarized in Table 2.

### 3.2. Quasi-Static Out-of-Plane Compression Tests

**Figure 4:**
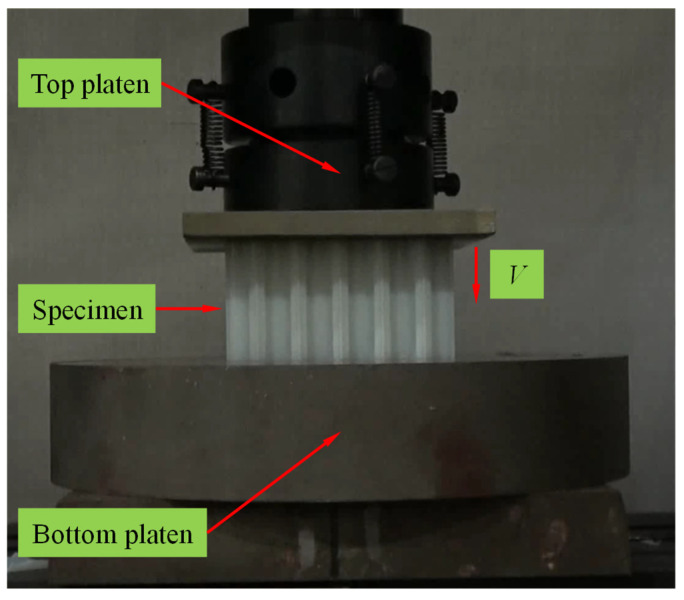
Quasi-static out-of-plane compression test setup for CHHH specimens.

According to the Chinese standard [45], quasi-static out-of-plane compression tests on the CHHH specimens were conducted using a universal testing machine, as shown in Figure 4. During each compression test, the specimen was positioned on the stationary bottom platen of the machine, while the top platen was moved downward at a loading rate of 1 mm/min to apply compression displacement. Both the force and displacement were recorded by the machine, and then they were converted into nominal stress–strain curves according to the geometric dimensions of the CHHH specimens. To ensure the reliability of the test data, three specimens were tested for each hierarchical parameter configuration of the CHHH. In addition, to obtain the deformation modes of the specimens, a video camera was adopted to record the entire test process.

## 4. Results and Discussion

### 4.1. Deformation Modes

**Figure 5:**
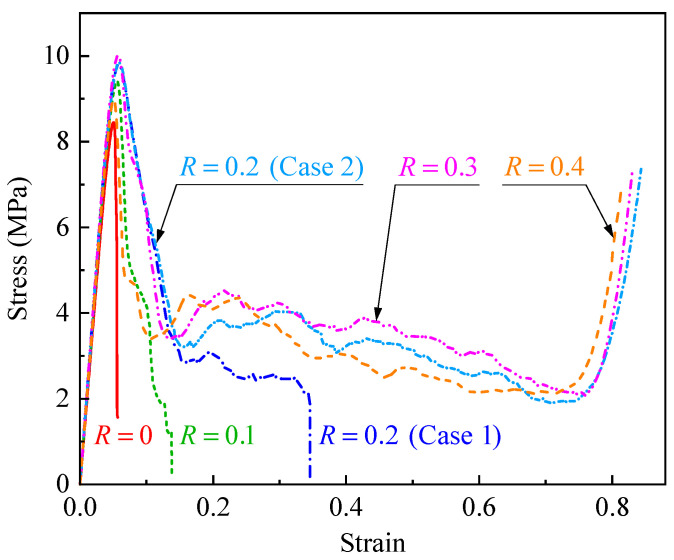
Stress–strain curves of CHHHs with different *R*.

Figure 5 presents the stress–strain curves of CHHHs with different hierarchical parameters *R*. It can be seen that the stress–strain curve of the RHH (namely a CHHH with R=0) firstly exhibited a linear elastic region, and then the stress decreased rapidly after reaching the initial peak stress. Additionally, the stress–strain curve of the CHHH with R=0.1 was similar to that of the RHH. For the CHHH with R=0.2, the stress–strain curves exhibited two different cases: the stress–strain curve of case 1 was similar to that of the RHH, while that of case 2 was similar to the stress–strain curve of regular metallic honeycombs under out-of-plane loading [6], firstly exhibiting a linear elastic region, followed by a plateau region, and finally a densification region where the stress increased rapidly. Note that the stress in the plateau region of case 2 decreased with increasing strain, while that of metallic honeycombs was nearly constant [6], and this was the difference between them. For CHHHs with R=0.3 and 0.4, their stress–strain curves also had a plateau region with gradually decreasing stress, which was similar to that of case 2 for the CHHH with R=0.2. The difference between the stress–strain curves of CHHHs with different *R* was caused by their distinct deformation modes.

**Figure 6:**
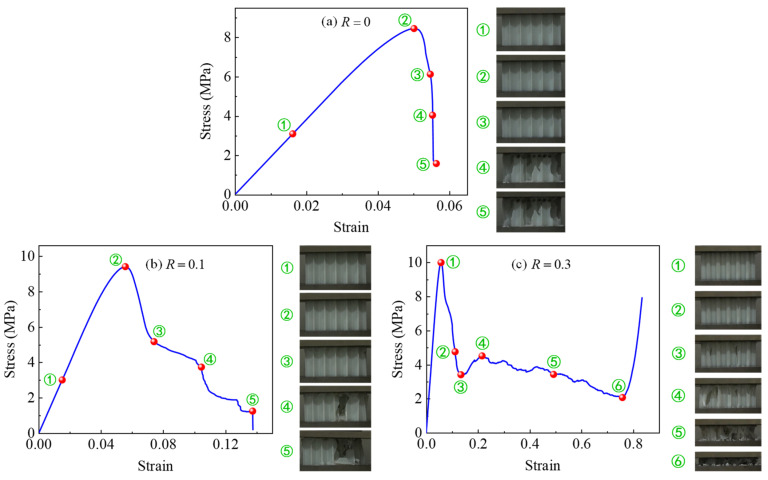
Deformation processes of CHHHs with (**a**) R=0, (**b**) R=0.1, and (**c**) R=0.3.

To analyze the deformation modes of CHHHs with different *R*, a sequence of the representative deformed configurations of CHHHs with R=0, 0.1, and 0.3, corresponding to the red dots on the stress–strain curves, is summarized in Figure 6. As illustrated in Figure 6a, for the RHH (CHHH with R=0), no significant deformation was observed in the linear elastic region, as shown in configuration ①. Upon reaching the initial peak stress, small local buckling of the cell walls was observed (configuration ②). Subsequently, the stress decreased rapidly, accompanied first by significant local buckling of the cell walls, followed by obvious fracture of the cell walls, and finally, most of the cell walls fractured, as shown in configurations ③, ④, and ⑤, respectively. The deformation process (Figure 6b) of the CHHH with R=0.1 was similar to that of the RHH, which accounts for the similarity observed in their stress–strain curves. However, a difference was that the fracture of the cell walls of the CHHH with R=0.1 was less pronounced and occurred later compared to that of the RHH.

The stress–strain curve and the corresponding deformed configurations of the CHHHs with R=0.3 are presented in Figure 6c. It can be seen that no obvious deformation was observed upon reaching the initial peak stress, as shown in configuration ①. Subsequently, the stress decreased rapidly, and at the stress corresponding to configuration ②, a fracture of the cell wall was observed at one of the circular vertices of the specimen for the first time. As the stress decreased to the first local minimum, the number of cell wall fractures increased to two, with the initial fracture becoming more severe, as shown in configuration ③. With continued compression, more cell wall fractures occurred, as shown in configurations ④ and ⑤, and the stress tended to decrease gradually. Nevertheless, it was interesting to observe that the stress began to increase rapidly upon reaching configuration ⑥, in which most of the cell walls came into contact with each other, leading to the specimen becoming stiffer and entering the densification region with increasing stress. Note that although the stress–strain curve of the CHHH with R=0.3 resembles that of the metallic honeycomb [6], the deformation modes are different. From the above results, it can be concluded that the deformation modes of CHHHs can be varied by changing *R*. The stress–strain curves (Figure 5) and deformation modes (Figure 6) indicate that elastic buckling dominates the compressive response of CHHH specimens, particularly for specimens with small *R*. Furthermore, compared to the catastrophic failure observed in the RHH and CHHHs with a small *R*, CHHHs with a large *R* tend to exhibit higher damage resistance and more stable stress responses. Therefore, the introduction of circular-vertex-based hierarchy can overcome the catastrophic failure of the RHH and contribute to a stable response.

### 4.2. Mechanical Properties

**Figure 7:**
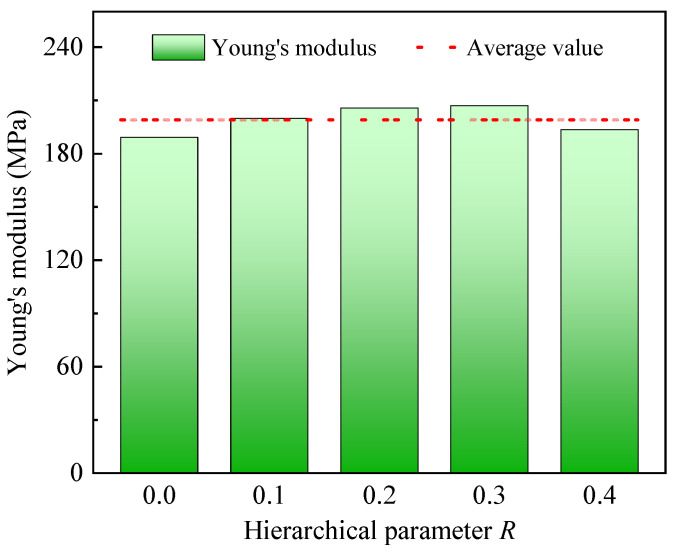
Young’s modulus of CHHHs with different *R*.

Young’s modulus is a crucial elastic mechanical property for materials, and it can be determined by the slope of the stress–strain curve within the linear elastic region. From Figure 5, it can be seen that the slopes in the elastic region for CHHHs with different *R* are almost the same. To show it more clearly, Young’s modulus $E_{3}^{*}$ of the CHHH was calculated and is presented in Figure 7. We found that Young’s moduli of CHHHs with different *R* fluctuate slightly around their average value, and the maximum error between the Young’s moduli and their average value is within 8.5%. The almost consistent $E_{3}^{*}$ can be explained by the finding that Young’s modulus of honeycombs under out-of-plane loading reflects Young’s modulus $E_{s}$ of base material, and the ratio $E_{3}^{*}/E_{s}$ is scaled by the relative density [46,47], which is a constant in this study.

**Figure 8:**
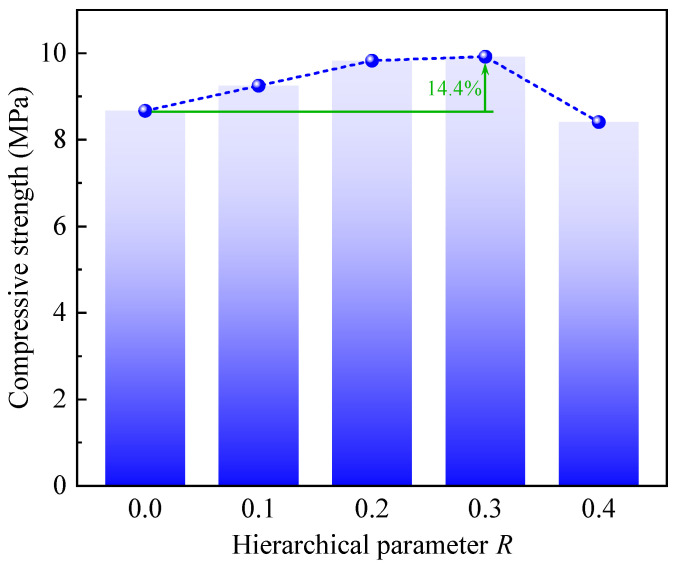
Compressive strength of CHHHs with different *R*.

Figure 8 shows the compressive strengths of CHHHs with different *R*, where the compressive strength represents the initial peak stress in the stress–strain curves (Figure 5). It can be seen that the compressive strength first increases slowly and then decreases quickly with increasing *R*, and the maximum value is achieved at R=0.3. The same variation trend and maximum point of compressive strength for CHHHs made of aluminum alloy 6061-T4 were also found in the study of Chen et al. [48]. Moreover, the compressive strength of the RHH is 8.67 MPa. The maximum compressive strength of the CHHH (R=0.3) is 9.92 MPa, which is 14.4% higher than that of the RHH with the same mass. Additionally, in the study of Chen et al. [48], the compressive strengths of the RHH and CHHH with R=0.3 were, respectively, 35.95 MPa and 38.78 MPa, and the maximum increase in compressive strength was 7.9%. Therefore, we surmised the increase in compressive strength of the CHHH when compared to that of the RHH is not significant.

**Figure 9:**
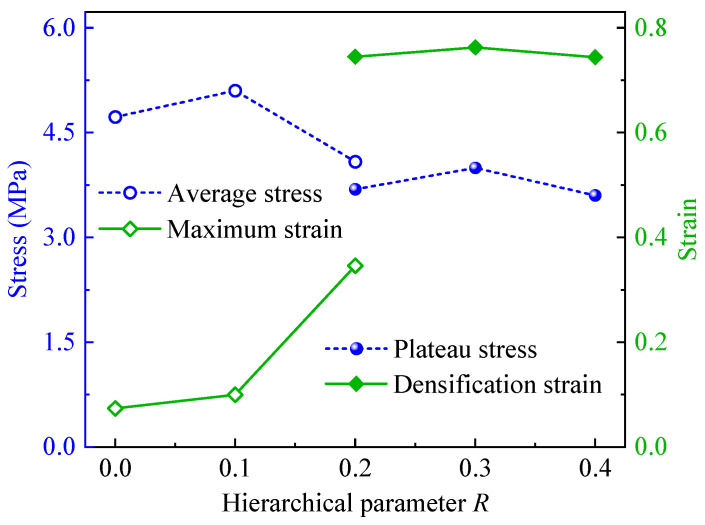
Average and plateau stresses and the corresponding strain of CHHHs with different *R*.

Figure 9 presents the average and plateau stresses and the corresponding strains of CHHHs with different *R*. Here, the average and plateau stresses are calculated by

(3) $$\sigma^{*}=\frac{1}{\varepsilon^{*}}\int_{0}^{\varepsilon^{*}}\sigma \left(\varepsilon \right)d\varepsilon$$

where $\varepsilon^{*}$ is, respectively, the maximum strain for CHHHs with R=0, 0.1, and 0.2 (case 1, Figure 5) and the densification strain for CHHHs with R=0.2 (case 2, Figure 5), 0.3, and 0.4. In this study, the densification strain is determined using the energy absorption efficiency method [49]. It can be seen from Figure 9 that with the increase in *R*, the maximum strain also increases. However, the maximum strain is small and does not reach the densification region. This is because the CHHHs with a small *R* fractured quickly and lost load-bearing capacity after reaching the initial peak stress, as shown in Figure 5 and Figure 6. As *R* continues to increase, the densification region can be achieved for CHHHs. Moreover, the average stresses of CHHHs with a small *R* are always higher than the plateau stresses of CHHHs with a big *R*. This is because the CHHHs with a big *R* have a long plateau region, with a plateau stress of about 40% of the corresponding initial peak stress. Additionally, both the plateau stress and densification strain reach their maximum values at R=0.3, leading to the highest energy absorption, as will be quantitatively discussed in the next subsection.

### 4.3. Energy Absorption Characteristics

**Figure 10:**
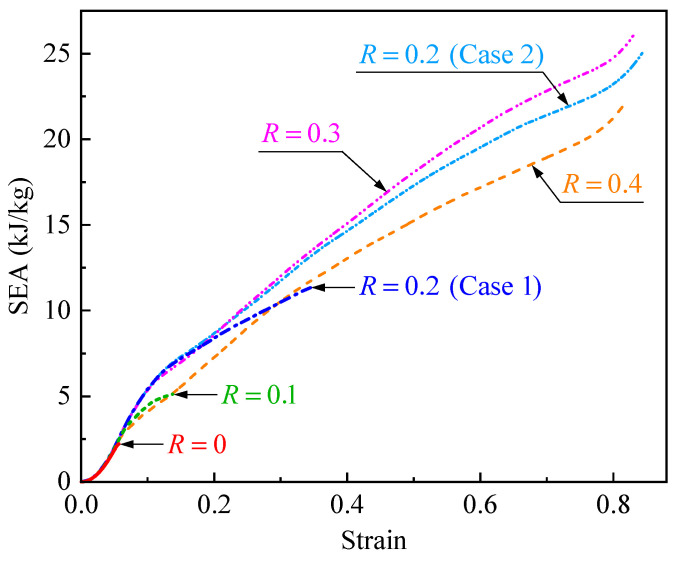
SEA–strain curves of CHHHs with different *R*.

**Figure 11:**
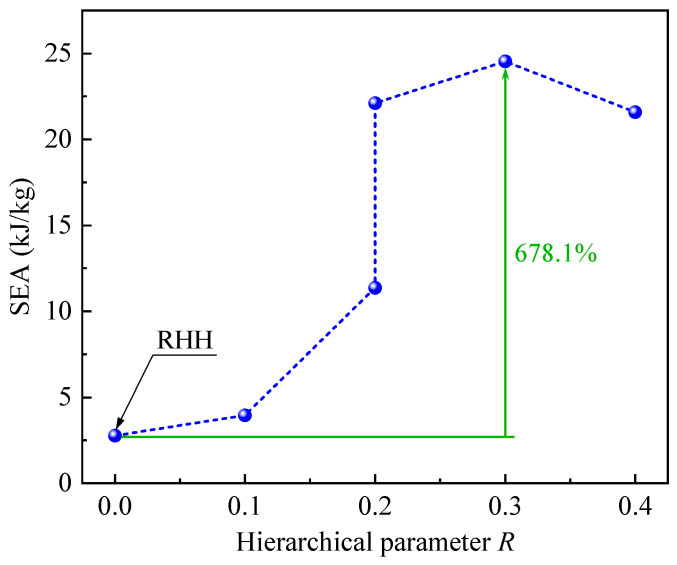
SEA of CHHHs with different *R*.

Specific energy absorption (SEA) is defined as the energy absorbed by materials or structures per unit mass, which is a crucial indicator for evaluating the energy absorption capability. Figure 10 shows the SEA–strain curves of CHHHs with different *R*, where the SEA is calculated by integrating the force–displacement curve and then dividing by mass. It can be seen that the SEA increases with increasing strain. Moreover, for CHHHs with *R* from 0 to 0.2, the maximum SEA also increases with increasing *R*. These maximum SEAs, along with the SEAs of CHHHs at the densification strain, are summarized in Figure 11. Here, the maximum SEAs are presented for CHHHs with R=0, 0.1, and 0.2 (case 1), while the SEAs at the densification strain are presented for CHHHs with R=0.2 (case 2), 0.3, and 0.4. As can be seen from Figure 11, the SEA first increases and then decreases with increasing *R*, reaching its maximum value at R=0.3. Additionally, the maximum SEA of CHHHs or the SEA of CHHHs at the densification strain is always higher than the maximum SEA of the corresponding RHH with the same mass. Notably, a quantitative comparison shows that the SEA of a CHHH with R=0.3 is 678.1% higher than that of the RHH. This indicates that the energy absorption capability of the RHH can be significantly enhanced by introducing a circular-vertex-based hierarchy and selecting an appropriate value of *R*.

## 5. Conclusions

In this study, polymeric circular-vertex-based hierarchical hexagonal honeycombs (CHHHs) with various hierarchical parameters were fabricated using a 3D-printing technique, and their quasi-static out-of-plane compressive mechanical behavior was experimentally investigated. The main conclusions are summarized as follows:

(1) Compared to a regular hexagonal honeycomb (RHH), the CHHH exhibited a superior mechanical performance, including higher damage resistance and strength, a more stable stress response, and enhanced energy absorption, primarily due to the introduction of circular-vertex-based hierarchy.
(2) The hierarchical parameter *R* has a significant effect on the deformation modes of CHHHs. The results indicated that the deformation processes of CHHHs with different *R* were accompanied by cell wall fractures, which led to catastrophic failure in CHHHs with small *R*. However, as *R* increased, the deformation and stress response of CHHHs became more stable.
(3) With the mass held constant, Young’s modulus of the CHHH remained nearly unchanged as *R* increased. In contrast, both the compressive strength and specific energy absorption (SEA) initially increased and then decreased, reaching their maximum values at R=0.3. Notably, the maximum compressive strength and SEA of the CHHH were, respectively, 14.4% and 678.1% higher than those of the RHH.

## Figures and Tables

**Table 1:** Geometric dimensions of CHHH specimens with different hierarchical parameters.

$\bar{\rho }$	*R*	*r* (mm)	*t*_1_ (mm)	*L_x_* (mm)	*L_y_* (mm)	*b* (mm)
0.1053	0	0	0.7500	76.21	72	40
0.1053	0.1	0.8	0.6408	76.21	72	40
0.1053	0.2	1.6	0.5321	76.21	72	40
0.1053	0.3	2.4	0.4562	76.21	72	40
0.1053	0.4	3.2	0.3997	76.21	72	40

**Table 2:** Basic mechanical parameters of VeroWhitePlus.

Density	Young’s Modulus	Poisson’s Ratio	Yield Strength	Ultimate Strength
1180 kg/m^3^	2.18 GPa	0.33	31.54 MPa	37.98 MPa

## Data Availability

The original contributions presented in the study are included in the article, further inquiries can be directed to the corresponding author.
